# Retraction Note: Using a new HSPC senescence model in vitro to explore the mechanism of cellular memory in aging HSPCs

**DOI:** 10.1186/s13287-023-03258-y

**Published:** 2023-02-28

**Authors:** Yongpin Dong, Chunni Guo, Wuxiong Zhou, Wenfang Li, Lina Zhang

**Affiliations:** 1 Institute of Basic Medicine, Shanghai University of Traditional Chinese Medicine, 1200 CaiLun Ave., Pudong, Shanghai, 201203 China; 2 Department of Emergency and Critical Care Medicine, Shanghai Changzheng Hospital, The Second Military Medical University, Shanghai, China; 3 Department of Neurology, Shanghai General Hospital, Shanghai Jiao Tong University School of Medicine, Shanghai, China

**Retraction to: Stem Cell Research & Therapy (2021) 12:444** **https://doi.org/10.1186/s13287-021-02455-x**

The Editors-in-Chief have retracted this article. After publication, concerns were raised regarding the authenticity of the presented data. The authors were unable to provide the raw data for western blot validation, and did not follow the journal's policy for RNA-Seq data sharing. Additionally, in Fig. 5c, the western blot for Ezh1 (lane 2) appears to overlap with Rae28 (lane 1). The Editors-in-Chief therefore no longer have confidence in the data presented in this article.

Yongpin Dong, Wenfang Li and Lina Zhang do not agree to this retraction. Chunni Guo and Wuxiong Zhou have not responded to any correspondence from the editor or publisher about this retraction.

